# Scientists on the spot: Relaxing the heart in hypertrophic cardiomyopathy

**DOI:** 10.1093/cvr/cvad049

**Published:** 2023-04-26

**Authors:** Monika M Gladka, Jolanda van der Velden

**Affiliations:** Department of Medical Biology, Amsterdam University Medical Center, Amsterdam Cardiovascular Sciences, Amsterdam, The Netherlands; Department of Physiology, Amsterdam UMC, Vrije Universiteit Amsterdam, Amsterdam Cardiovascular Sciences, Location VUmc, O2 Science building, De Boelelaan 1108, 1081HZ Amsterdam, The Netherlands

**Keywords:** Hypertrophic cardiomyopathy, HoCM, Genetic heart disease, HFpEF, Myosin inhibitor

**Figure:**
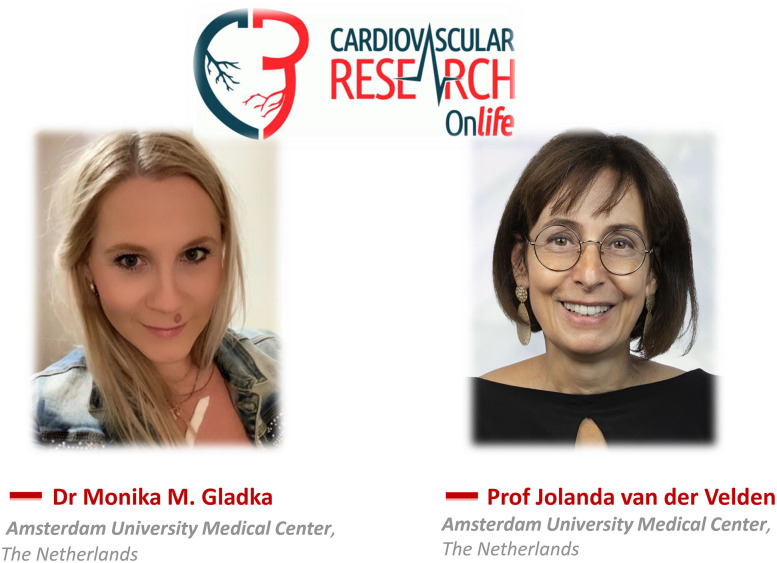
Dr Monika M. Gladka, from Amsterdam University Medical Centre (The Netherlands), interviews Prof. Jolanda van der Velden, Director of the Amsterdam Cardiovascular Sciences Institute (The Netherlands).

**Highlight:** In this *Cardiovascular Research* On*Life* interview, Professor van der Velden discusses recent advancements that are crucial for treatment of inherited cardiomyopathies in the future, as well as the challenges in creating effective therapies.

## Summary of interview

Prof. van der Velden discusses a recent clinical trial which used a myosin inhibitor to treat patients with obstructive hypertrophic cardiomyopathy (HoCM). The most frequent cause of HCM is mutations in the heart muscle proteins of the sarcomere, which disrupt the super-relaxed (SRX) state of the myosin heads. SRX is an energy-saving state, and it is beneficial for the heart. Recent experimental research and the clinical trial showed that myosin inhibitors could counteract the detrimental effect of the mutation and bring the heart back to the relaxed, low-energy-consuming state, which is a promising outcome. Prof. van der Velden explains that effective treatments to prevent the development of genetic heart disease are still lacking. The only treatments we have are mainly symptomatic, and therefore, there is still a need for new therapies. Another aspect that needs improvement, highlighted by Prof. van der Velden, is the prognosis of the disease. There are many mutation carriers, but not all of them will develop cardiac disease and arrhythmias. We need to understand better who is at risk and why.

When asked about her achievements, Prof. van der Velden mentioned a technology she set up with Prof. Ger Stienen in Amsterdam and a study where she collaborated with Prof. Walter Paulus some years ago to measure function in human cardiac biopsies contributing to identifying key disease mechanisms. This method revealed increased stiffness of cardiac muscle cells as cause of impaired relaxation in Heart Failure with Preserved Ejection Fraction (HFpEF) patients.

Lastly, she advises young researchers that the key to success is to be themselves and establish themselves as individuals with unique expertise.

## Authors

**Biography:** Dr Monika Gladka is currently an Assistant Professor at University Medical Center in Amsterdam. Her current research focuses on understanding the molecular mechanisms that regulate cardiac repair, intending to identify new players to develop novel, improved gene therapies. She uses several state-of-the-art techniques such as single-cell sequencing, enabling an in-depth mechanistic understanding of the biological processes in injured cardiomyocytes. In 2016 and 2020, she received two prestigious Dr E. Dekker personal grants from the Dutch Heart Foundation for heart repair research. She is actively involved in several cardiac societies acting as a board member of Young@Heart from the Netherlands Heart Institute and a nucleus member of the Scientists of Tomorrow from the European Society of Cardiology.

**Biography:** Professor Jolanda van der Velden is Chair of the Department of Physiology at Amsterdam University Medical Center and Director of the Amsterdam Cardiovascular Sciences Institute in the Netherlands. Her research interest involves investigating changes in cellular protein composition and function, which contribute to impaired cardiac muscle performance in heart failure and could be a target for drug therapy. Prof. van der Velden is a member of several consortia and is internationally recognized for her work on genetic heart disease. She has published over 230 papers in peer-reviewed, international journals.

